# Ecofriendly Usability of Mushroom Cultivation Substrate as a Ruminant Feed: Anaerobic Digestion Using Gas Production Techniques

**DOI:** 10.3390/ani12121583

**Published:** 2022-06-19

**Authors:** Valiollah Palangi, Adem Kaya, Ali Kaya, Ilias Giannenas

**Affiliations:** 1 Department of Animal Science, Agricultural Faculty, Ataturk University, 25240 Erzurum, Turkey; akaya@atauni.edu.tr (A.K.); alikaya@atauni.edu.tr (A.K.); 2 Laboratory of Nutrition, Faculty of Veterinary Medicine, Aristotle University of Thessaloniki, 54124 Thessaloniki, Greece

**Keywords:** mushroom-cultivated substrate, agro-industrial by-products, in vitro digestibility, methane emission

## Abstract

**Simple Summary:**

Ruminants might use process and agricultural by-products to meet their maintenance, growth, and production needs. Generally, feed is the major cost for an animal farm, yet utilization of agro-industrial waste (such as mushroom cultivation substrate) not only reduces these expenses, but could also help with the issue of environmental pollution. Accordingly, mushroom cultivation waste might be used for animal feeding after harvesting, because of its good-quality substrate, which is beneficial for ruminants. Therefore, the objective of this study was to evaluate the mushroom-cultivated substrate microscopic surface and its fermentability. Mushroom cultivation led to lower ether extract, acid detergent fiber, and crude fiber level of substrate. Our results show that biological treatment could reduce fiber components while increasing feed digestibility, providing new insight into the use of biological pretreatment to produce ruminant feed. According to the results, biological processing of mushroom cultivation substrate might increase in vitro fermentation. In conclusion, mushroom-cultivated substrate might be used as feed in ruminant diets.

**Abstract:**

The current study was carried out to evaluate the nutritive value of mushroom-uncultivated and -cultivated substrates, and their in vitro gas and methane production. The experiment was conducted in a completely randomized design, and analyzed with GLM using SAS 9.4. Analysis of the structural morphology of mushroom-cultivated substrate was performed using a scanning electron microscope. Mushroom cultivation led to lower ether extract, acid detergent fiber, and crude fiber level of substrate (*p* < 0.05). Mushroom-cultivated substrate showed higher in vitro cumulative gas production (*p* < 0.05). Moreover, mushroom cultivation led to a higher sample surface, and improved the microorganisms’ access to feed materials, thus stimulating rumen fermentation and increasing methane production (*p* < 0.05). The organic matter digestibility, metabolizable energy, and net energy lactation values were higher for mushroom-cultivated substrate than uncultivated substrate. The results demonstrate that mushroom-cultivation not only increases the contact surface of cellulose, leading to higher ruminal microorganisms’ access to feedstuff, but could also had higher nutritive value; this material might be used in ruminant ration formulation, to reduce environmental pollution and feed costs.

## 1. Introduction

An animal’s response to nutrients in terms of the quantitative aspects of digestion and metabolism is an important detector by which to evaluate ruminant feed [1]. Digestibility and rumen degradability are parameters that play an important role in determining feed quality. Fermentation characteristics of feedstuffs in rumen fluid might be studied using in vivo, in situ, and in vitro techniques [1]. The in vitro gas production system helps to better quantify nutrient utilization, and its accuracy in describing digestibility in animals has been validated in numerous experiments [2].

The growing human population has highlighted the importance of the need to provide food for the growth and development of human societies. The relationship between food supply and development is very clear, and by developing the food supply chains, we acquire both the morphology and the dynamics of these factors; this is possible with the optimal use of food resources. Ruminants have a high ability to convert low-quality feed into high-quality human-edible products. Proper nutrition management of dairy cows in industrial farms has a very important role in the production and economic outcome of the herd. Ruminant animals need to obtain optimum efficiency from the amount of feed that they consume, which can be achieved if they are fed the right amount and type of feed [3]. Serrapica et al. [4] demonstrated that cakes of tobacco and hemp have potential as alternative protein supplements for ruminants.

Nowadays, high feeding costs are one of the major problems of livestock farms. Farmers often have to reduce the numbers of animals to cut the cost of feedstuffs due to the high price of commercial forages [5,6]. Ruminants might use process and agricultural by-products to meet their maintenance, growth, and production needs [7,8,9]. Hence, they recycle these residues back into the food chain. Generally, the price of feed is the major cost variable for an animal farm, and utilization of agro-industrial waste (such as mushroom cultivation spent substrate) as alternative feedstuffs, might help to reduce both feeding cost and environmental pollution. One of the agricultural wastes is the mushroom cultivation spent substrate, which is considered as an undesirable by-product in edible mushroom production centers, and is often removed by various methods, such as incineration or disposal. Substrates left over from mushroom cultivation is rich in microorganisms, extracellular enzymes, and nitrogen, which is thought to have the potential to be used as animal feed [10]. Mushrooms are grown in a substrate called compost, mostly composed of lignocellulosic material containing cellulose, lignin, and a small amount of protein [11]. Disposal of used compost at the end of mushroom cultivation is not only costly in terms of production units, but also causes environmental problems. During the growth of mushrooms, numerous active enzymes, capable of degrading lignin and improving the digestibility of substrate, are secreted by white-rot fungi mycelia [12]. The usefulness of enzymes in feed digestion has also been highlighted by Iannaccone et al. [13]. Additionally, mushroom-cultivated substrate is rich in terms of bioactive components, such as polysaccharides, vitamins, and some trace elements [14], which are beneficial for rumen fermentation processes. Mhlongo et al. [15] concluded that treating grape pomace waste with oyster mushroom spawn reduced fiber levels and increased crude protein content. Similar results were reported by Ahmed et al. [16], where fungal treatment of wheat straw increased dry matter (DM) and crude protein (CP) digestibility. Scanning electron microscopy (SEM) has made it possible to examine the surface and interior microstructure of feed. Scanning electron microscopy might offer a new perspective on digestion, and the parameters that enhance digestibility, helping to observe uneven surfaces that result from processing. Hence, we hypothesized that treatment with mushroom-cultivation may increase substrate contact surface, leading to higher ruminal microorganism access, hence improving ruminal degradability and nutritive value. Therefore, the objective of this study was to evaluate the effects of mushroom-cultivation on its microscopic surface, and its ruminal fermentability.

## 2. Materials and Methods

This study neither involved animal participation, experiment, nor animal tissue. Therefore, there was no need for approval from the ethics committee.

### 2.1. Mushroom Cultivation Spent Substrate

Mushroom-cultivated substrate was collected from a mushroom farm in Erzurum. Mushroom-uncultivated substrate (without *Agaricu bisporus* spores) and -cultivated substrate (contains *Agaricu bisporus* spores) was considered. Two types of samples (two treatments containing mushroom-cultivated and non-cultivated substrates; six replicates (each sample contains 0.2 g) for each treatment in each incubation time) were randomly collected from different levels of the shelves at different areas of the cultivation room, and dried at room temperature to prepare air-dried samples. Subsequently, each sample was milled with an appropriate sieve.

### 2.2. Scanning Electron Microscope Analysis

Scanning electron microscope analysis involves surface scanning by an electron beam in order to examine the structures of the samples under investigation. Scanning electron microscope analysis of the structural morphology of mushroom-cultivated substrate was performed using the Zeiss Sigma 300 SEM device, which is in the inventory of Atatürk University Eastern Anatolia Advanced Technologies Application and Research Center in Turkey. To ensure the conductivity of the sample, it was coated with gold. The sample was then placed in the SEM unit, and the system was placed in a vacuum. When the vacuum reached a certain value, adjustments were made according to the voltage value determined from the sample properties, and the device was set to a high voltage. The image formed as a result of the interaction of the electron beam and the sample was followed on the screen, and the sample holder moved along the X, Y, and Z axes to find the region to be examined, and the sharpening and focusing settings were adjusted to the desired magnifications.

### 2.3. Chemical Analyses

Proximate analysis of the mushroom cultivation substrate samples (before and after mushroom cultivation), including dry matter (DM) and crude protein (CP), ether extract (EE) and ash, was conducted according to AOAC [17]. The Kjeldahl method was utilized to determine the N content of samples (AOAC, 2005; Method 984.13). The method of Van Soest et al. [18] was used to determine the acid detergent fiber (ADF) and neutral detergent fiber (NDF) of samples. Crude fiber was analyzed by AOAC 978.10.

### 2.4. In Vitro Gas Production

Rumen fluid was collected from the newly slaughtered cattle (Holstein Friesian) for in vitro gas production measurements (as soon as the animals were slaughtered), according to the method reported by Palangi et al. [19], and transferred to the laboratory under anaerobic conditions. After transport, the sample was mixed and blended under a CO_2_ headspace for 30 s to remove any additional particles and/or attached organisms, and percolated through a four-layer cheese cloth to a flask that had been warmed at 39 °C, and promptly taken to the laboratory. Strained ruminal inoculums were mixed thoroughly at 39 °C, together with the synthetic saliva (30 mL, in a ratio of 1:2 (ruminal fluid: buffer)), to produce a homogeneous digestion medium. Approximately 0.2 g of ground mushroom-uncultivated and -cultivated substrate was incubated in rumen fluid in 100 mL calibrated glass syringes to determine in vitro gas production using the method of Menke and Steingass [20]. To standardize samples and correct the gas production with the ruminal fluid origin, six syringes containing only ruminal fluid and buffer solution mixture without feed sample were considered as blank. Gas volume of feed samples were measured at 6, 12, and 24 h of incubation. Gas produced was measured after the correction of the estimated gas production with 49.61 mL per 0.2 g on dry matter bases, which were reported as the standard by Hohenheim University [21]. The amount of methane gas at 6, 12, and 24 h of in vitro incubation was measured using an infrared CH_4_ analyzer (Sensor Europe GmbH, Erkrath, Germany). Methane production (mL) was computed as follows:

CH_4_ production (mL) = Total gas production (mL) × percentage of CH_4_ (%)

### 2.5. OMD, ME, and NEL Content

Metabolizable energy (ME) and organic matter digestibility (OMD) of raw feed materials were determined by the following equations reported by Menke and Steingass [20]. Net energy lactation (NEL) was calculated using the equations reported by Palangi [22].

OMD % = 15.38 + 0.8453 × GP + 0.0595 × CP + 0.0675 × Ash

ME, MJ/kg DM = 2.20 + 0.1357 × GP + 0.057 × CP + 0.002859 × EE^2^

NEL (MJ/kg KM) = 0.101 × GP + 0.051 × CP + 0.112 × EE

(GP: 200 mg gas production of 24 h, CP (%) and EE (%)).

### 2.6. Statistical Analysis

Data were subjected to variance analysis with GLM using SAS 9.14 [23] package program. Duncan’s test was used to compare the means of the groups.

Obtained data were analyzed according to statistical model:

Y_ij_= μ + T_i_ + e_ij_

where, Y_ij_ is the dependent variable, μ is the overall mean, T_i_ is the effect of cultivation, and e_ij_ is the random error.

## 3. Results and Discussion

### 3.1. SEM Analysis of Mushroom Substrate

**Figure 1:**
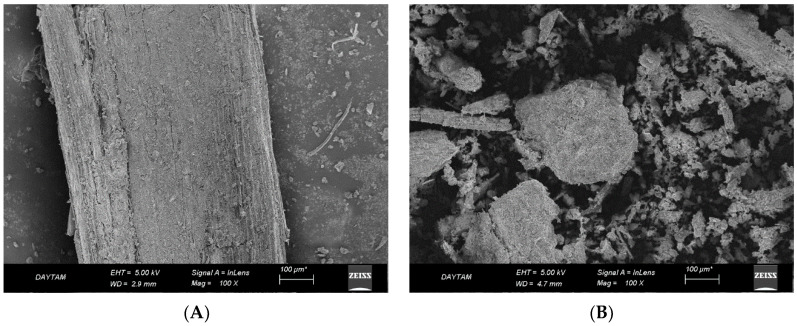
SEM images of mushroom-uncultivated (**A**) and -cultivated (**B**) substrate.

Scanning electron microscope images of mushroom-uncultivated and -cultivated substrate are shown in Figure 1A,B, respectively. As illustrated in Figure 1, the cultivation of substrate with mushrooms led to the crushing of the substrate material cell wall. As a result, the contact surface of cellulose was increased, leading to higher ruminal microorganism access to feed materials, hence improving ruminal degradability. As illustrated in Figure 1, mushroom processing resulted in a smaller particle size compared to uncultivated substrate. As seen in the figure, cracked areas in mushroom-cultivated substrate may facilitate colony development of the microorganisms, thus stimulating rumen fermentation. In agreement, de Lima Valença et al. [24] noted a direct relationship between the contact surface of microorganisms and ruminal retention time.

In accordance with our results, Shirmohammadi et al. [25] stated that more broken edges (larger particle size) not only provide greater surface areas for microorganisms to inhabit, but also allow them to develop colonies and digest the feedstuff.

### 3.2. Chemical Analyses

The obtained data for chemical composition are shown in Table 1. Mushroom enhancement decreased the EE, ADF, and CF level of the substrate. Our results show that biological treatment could reduce fiber components while increasing feed digestibility, and provide new insight into the use of biological pretreatment to produce ruminant feed [26]. Our substrate sample was comparable to the barley straw composition in the study of Ribeiro et al. [27] in terms of ADF values. The CP values of feed were comparable to the findings of Marino et al. [28] and Mhlongo et al. [15], yet higher than those reported by Nasiri et al. [29] and Yousefi [30], and lower than in Mahfuz et al. [31]. The obtained NDF and ADF values were higher than the findings of Kalvandi et al. [10], Dilfy et al. [32], and Yousefi et al. [33], and lower than those of Astudillo-Neira et al. [34]. Fazaeli and Masoodi [35] reported an increase in crude protein and ash in oyster mushroom-cultivated substrate. Our results agree with those of Fazaeli and Masoodi [35] in terms of ash quantity. High levels of ash in samples is normal since the substrate had to be mixed with soil prior to mushroom cultivation. Additionally, the high amount of ash in the cultivated treatments may be related to consumed organic matter by the fungus. In confirmation of this issue, Kalvandi et al. [10] reported that soil is one of the main components in the preparation of compost for mushroom cultivation, and soil contamination in food causes the percentage of raw ash to increase, and consequently, the percentage of organic matter to decrease. To date, studies have reported variable chemical compositions of mushroom-cultivated substrate; different geographical areas, processing types, cultivation, and climate differences, as well as conservation systems seem to be the reason for this variation [21].

### 3.3. In Vitro Gas and Methane Production

As shown in Table 2, mushroom-uncultivated and -cultivated substrate had the highest cumulative gas and methane production (*p* < 0.05). The volume of gas produced increases as it approaches the end of the incubation time because, in the gas production test method, the microbial population is still constant at all hours of incubation, and there is no entry or exit routes for microorganisms. Similar results were reported by Mhlongo [36], where mushroom spawning of red grape pomace linearly increased the rate of in vitro gas production. In the current experiment, the amount of cumulative gas produced by fermentation in vitro were lower than the results of Kalvandi et al. [10] and Baziuon et al. [37]. The amount of gas production in the early stages is due to the differences in the level of nonstructural carbohydrates (NSC), such as sugars, pectin, and starches, that rapidly ferment and produce gas [38]. The effect of microbial enzymes on feedstuff depends on the nature of the feed and its availability to microorganisms. Mushroom cultivation increases the sample surface, improving the microorganisms’ access; therefore, rumen fermentation increases and gas and methane production increases. In agreement, Mhlongo [36] noted that lignolytic activity of oyster mushroom spawn increases the accessibility of cellulose and hemicellulose to rumen microbes. Budzianowski [39] reported that fiber treatment might reduce the crystallinity of cellulose as well as the lignin content, facilitating the entry of microorganisms. Likewise, Valadares et al. [40] indicated that the white-rot fungi biodegradation process would hydrolyze beta-1, 4-glycosidic linkage bonds, therefore degrading cellulose and the hemicellulose matrix by cellulases. The hemicellulose is then degraded into monosaccharides and acetic acid by hemicellulases through the formation of oligosaccharides from xylan [41], resulting in biodegradation by white-rot fungi. Consistent with our results, Fan et al. [42] stated that treating rice straw as mushroom spent substrate could substantially enhance its feed value. Yousefi et al. [33] concluded that button mushroom stipe might be substituted into the ruminant diet without adversely affecting the health and performance of animals. The in vitro gas production is mainly from two sources, carbohydrates (carbon dioxide and methane), obtained directly from microbial fermentation and indirectly by buffering short-chain fatty acids (carbon dioxide released from bicarbonate buffer). However, changes in the microbial activity of ruminal fluid may affect the fermentation rate. In general, the most important factors affecting gas production are harvest time, amount of water-soluble and -insoluble carbohydrates, amount of NDF, the origin of the collected microbial fluid, species of the ruminal fluid donor animal, rumen fluid collection time, and the basal ration of the ruminal fluid donor animal. According to Menke and Steingass [20], gas production is merely affected by chemical composition and physical properties of the feed. Nevertheless, mushroom processing has increased the availability of nutrients, thus increasing gas production. Moreover, it is possible that lignin metabolites, which inhibit the ruminal microorganisms and/or their functional enzymes, are broken down by fungal enzymes, thus losing their inhibitory properties. Therefore, higher fermentation capacity increases the gas production in mushroom-treated samples.

Ruminal anaerobic fermentation of nutrients is a common pathway by which to reduce carbon dioxide and hydrogen ions (H^+^) to methane [19]. Eructed CH_4_ has been estimated to account for 2–12% of ingested feed energy [21], and excess hydrogen is removed from the reduction of nicotinamide adenine dinucleotide (NAD^+^) by the formation of methane by methanogens. Therefore, due to higher methane production in mushroom-cultivated substrate, part of the energy is wasted, thus other processing methods should be used to reduce the availability of hydrogen and production of methane.

### 3.4. OMD, ME, and NEL Contents

**Figure 2:**
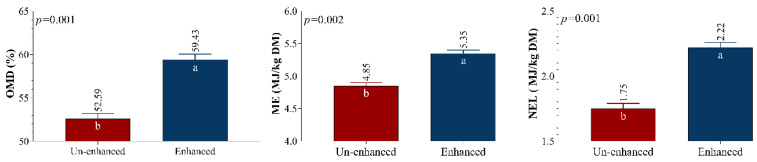
Organic Matter Digestibility (OMD), Metabolic Energy (ME), and Net Energy Lactation (NEL) content of mushroom-uncultivated and -cultivated substrate.

The OMD, ME, and NEL contents of mushroom-uncultivated and -cultivated substrates are presented in Figure 2. As illustrated, mushroom processing resulted in a significant increase in OMD, ME, and NEL (*p* < 0.05). Given that the total amount of produced gas during 24 h of incubation was utilized to calculate metabolic energy and OMD values, increased fermentation in cultivated substrate led to increased gas production, and thus indirectly affected OMD, ME, and NEL contents. Our OMD results agree with those reported by Baziuon et al. [37]; however, our ME and NEL levels were lower than those of the same study. Mushroom enhancement might reduce the lignin content by reducing the crystallinity of cellulose, thus increasing the nutritional value.

The obtained results are consistent with the findings of Barnes et al. [43] regarding the importance of the chemical constituents of feed, and their interactions, for the availability of feed materials to ruminants. In addition, OMD was higher in the mushroom cultivation substrate. The total amount of daily gas produced during 24 h of incubation was utilized to calculate the OMD value. The high in vitro gas production values of treatment containing cultivated substrate led to greater levels of OMD.

Due to its high ash content compared to the mushroom-uncultivated substrate, and given that this is one of the parameters involved in the relative equation, it has probably indirectly affected the value. The chemical composition (cellulose, hemicellulose, and lignin ratios) affects NDF digestibility [38]. Accordingly, high NDF and low ADF levels of mushroom-cultivated substrate indirectly leads to greater energy content (ME and NEL).

## 4. Conclusions

According to the evaluation of nutrient compositions, the amounts of ME, and specific NEL and OMD content of mushroom-cultivated substrate, this by-product has a higher nutritional value compared to the uncultivated substrate. Additionally, due to the acceptable level of protein in mushroom-cultivated substrate, and its low levels of ME, it might be used to reduce the price of ruminant rations using nutritional measures and proper synchronization with energy sources. Agro-industrial by-products could potentially be sustainable feedstuffs, yet the practicability of these materials needs to be studied for their proper application by the livestock sector. Usability of mushroom-cultivated substrate as feedstuff and bio-recycling might reduce pollution, while helping with food security. We recommend mushroom-cultivated substrate with higher nutritional value as inexpensive feedstuff in ruminant diets.

## Figures and Tables

**Table 1:** Chemical composition of mushroom-uncultivated and -cultivated substrate.

Feed	DM	Ash	CP	EE	NDF	ADF	ADL	CF
Uncultivated substrate	33.11	34.42 ^b^	10.46	12.29 ^a^	54.39	44.50 ^a^	27.53	13.33 ^a^
Cultivated substrate	33.35	38.04 ^a^	10.60	9.35 ^b^	57.54	39.99 ^b^	29.70	8.26 ^b^
SEM	0.36	0.53	0.15	0.03	0.76	0.48	0.60	0.43
*p* value	0.752	0.026	0.688	<0.0001	0.108	0.010	0.145	0.004

^a, b^: Differences between the averages indicated by different letters in the same column are significant (*p* < 0.05). SEM = Standard Error Means.

**Table 2:** In vitro gas and methane production of mushroom-uncultivated and -cultivated substrate (mL/200 mg DM).

Feed	Incubation Times (Gas) (h)			Methane (mL)			pH		
	6	12	24	6	12	24	6	12	24
Uncultivated substrate	3.98 ^b^	7.29	11.94 ^b^	0.75 ^b^	0.48 ^b^	0.81 ^b^	6.58	6.61	6.73
Cultivated substrate	6.96 ^a^	9.95	16.91 ^a^	1.14 ^a^	0.91 ^a^	1.24 ^a^	6.60	6.59	6.69
SEM	0.33	1.65	0.5	0.01	0.02	0.01	0.00	0.00	0.00
*p* value	0.003	0.065	0.001	0.006	0.023	0.009	0.184	0.170	0.115

^a, b^: Differences between the averages indicated by different letters in the same column are important. SEM = Standard Error Means.

## Data Availability

The datasets generated during and/or analyzed during the current study are available from the corresponding author (V.P.) on reasonable request.
